# Understanding care needs of cancer patients with depressive symptoms: The importance of patients' recognition of depressive symptoms

**DOI:** 10.1002/pon.5779

**Published:** 2021-08-10

**Authors:** Esmée A. Bickel, Anouk M. Auener, Adelita V. Ranchor, Joke Fleer, Maya J. Schroevers

**Affiliations:** ^1^ Department of Health Psychology University Medical Center Groningen University of Groningen Groningen the Netherlands

**Keywords:** cancer, common‐sense model, depressive symptoms, illness perceptions, oncology, psychological care needs, psycho‐oncology

## Abstract

**Objective:**

The majority of cancer patients with depressive symptoms does not perceive a need for psychological care. Reasons for this are still unclear. We examined the mediating role of cancer patients' perceptions of depressive symptoms in the relationship between depressive symptoms and perceived need for psychological care.

**Methods:**

For this cross‐sectional study, we recruited 127 Dutch cancer patients with moderate to severe levels of depressive symptoms (Patient Health Questionnaire [PHQ]‐9≥10) who did not receive professional psychological care. Depressive symptoms were measured with the PHQ‐9 questionnaire, by using three different depression score operationalizations. We used mediation analyses to test the mediating role of patients' illness perceptions (measured with subscales of the Brief Illness Perception Questionnaire) in the relation between depressive symptoms and need for care.

**Results:**

Whilst results did not show significant direct associations between depressive symptoms and perceived need for psychological care, we found positive indirect effects of severity (*B* = 0.07, SE = 0.04, *p* < 0.02), meeting the DSM‐5 diagnosis (*B* = 0.45, SE = 0.26, *p* < 0.02) and having relatively more affective symptoms (*B* = 2.37, SE = 1.10, *p* < 0.02) on need for care through the identity perception.

**Conclusions:**

Including assessments of patients' recognition of depressive symptoms and their perceptions of depression treatment efficacy might improve depression screening in cancer patients by more accurately identifying those with a need for psychological care. Moreover, improving patients' knowledge and recognition of symptoms as being depressive symptoms might be a possible target point in increasing care needs and hereby optimizing the uptake of psychological care in cancer patients with depressive symptoms.

## 1 BACKGROUND

Depressive symptoms are common in cancer patients: prevalence rates range between 8 and 36%.^1^, ^2^ Untreated, these symptoms can affect cancer patients' quality of life, treatment adherence and mortality.^3^, ^4^, ^5^ Screening programs can refer patients with high depressive symptom levels to psychological treatment. However, only half of cancer patients who screen positive for depressive symptoms will consequently engage in psychological treatment,^6^, ^7^, ^8^ mainly because perceived need for psychological care is low.^8^, ^9^, ^10^ While acknowledging patients' free choice, specific beliefs about symptoms or treatment might withhold patients from having a need for psychological care. This study therefore examined the role of illness perceptions about depressive symptoms in the relation between depressive symptoms and perceived need for care in cancer patients with depressive symptoms who currently do not receive psychological care.

The Common Sense Model of Self‐Regulation (CSM) is a well‐known theory to explain help‐seeking behaviors and states that patients form illness perceptions (e.g., consequences, duration or perceived control) as a response to the symptoms they perceive, which influence coping responses, which in turn influence illness outcomes.^11^ The CSM has been used extensively to examine cancer patients' perceptions of *cancer* (e.g., Richardson et al.^12^), but not their perceptions of *depressive symptoms,* even though it can be extended from physical conditions to mental health issues.^13^, ^14^ Besides being mediated by coping responses, illness perceptions can also directly influence illness outcomes. For instance, positive perceptions of treatment efficacy, expecting longer duration, foreseeing negative consequences and having a stronger understanding of depression were related to stronger help‐seeking behavior according to a systematic review.^15^

For patients with cancer *and* depressive symptoms, the role of depressive symptom perceptions in determining perceived need for psychological care has been neglected up to now. Cancer patients might perceive depressive symptoms differently than patients in primary care due to overlap between depressive symptoms and symptoms related to cancer (e.g., fatigue, appetite changes). Thus far, qualitative studies found that higher recognition of depressive symptoms and not considering symptoms as a normal part of life was related to higher need for psychological care in cardiovascular diseases,^16^ diabetes^17^ and other chronic illnesses.^18^

Depressive symptoms are highly heterogeneous (e.g., depressed mood and loss of interest, but also fatigue), and assessment of these symptoms in cancer patients can be difficult.^19^ This study will therefore focus on three operationalizations of depressive symptoms. Most studies use a sum score to measure severity of depressive symptoms^15^ but this does not account for the somatic overlap of depression and cancer.^19^, ^20^ A second, clinically relevant option is classifying patients as depressed or non‐depressed, based on the DSM‐5 diagnostic‐algorithm.^21^ This accounts for somatic overlap in symptoms since the two core symptoms of depression (i.e., depressed mood and loss of interest) need to be present. A third option is to focus on the *type* of depressive symptoms. When mainly experiencing somatic and few cognitive‐affective symptoms of depression, patients might attribute these symptoms to cancer and not to depression and thus not perceive a need for psychological support.^18^, ^22^, ^23^

The current study is the first to investigate whether perceptions of depressive symptoms explain an association between depressive symptoms and need for psychological care in cancer patients who currently do not receive psychological care for their elevated depressive symptoms. This leads to the following research questions: (1) To what extent are the three operationalizations of depressive symptoms related to need for psychological care?; (2) To what extent are the operationalizations of depressive symptoms related to perceptions of these symptoms?; (3) To what extent are perceptions of depressive symptoms related to need for psychological care?; and (4) Do perceptions of depressive symptoms mediate the relation between the operationalizations of depressive symptoms and need for psychological care?

Although previous literature shows mixed results, based on a large body of evidence we expect that severity of depressive symptoms and meeting the DSM‐5 diagnosis of depression will not be related to need for psychological care.^24^, ^25^, ^26^ We expect affective symptoms to relate to psychological care needs due to stronger attribution to depression.^18^, ^22^, ^23^ Based on the CSM, we expect that depressive symptoms will relate to patients' illness perceptions of these symptoms, and that these perceptions will be related to the need for psychological care—as is also shown by previous empirical research (e.g., Baines and Wittkowski^15^; Elwy et al.^27^)—and mediate the relation between depressive symptoms and need for psychological care.

## 2 METHODS

### 2.1 Study design

The current study included baseline data from a longitudinal observational study within a larger project examining psychological care needs in cancer patients with depressive symptoms. Data were obtained using self‐report questionnaires. The study was approved by the Medical Ethical Committee of the University Medical Center Groningen (2017/064).

### 2.2 Respondents

The target population comprised Dutch cancer patients who^1^: were 18 years or older,^2^ received any type of cancer diagnosis in the past 5 years, and^3^ showed moderate to severe levels of depressive symptoms (Patient Health Questionnaire [PHQ]‐9 ≥ 10). Patients were excluded if they received any type of professional psychological care at the moment of inclusion.

### 2.3 Procedure

Kantar Public*,* an international research agency with a respondent panel available for research (https://www.kantar.com/), carried out recruitment of respondents. Respondents in their database were contacted by e‐mail and asked if they had received any type of cancer diagnosis in the past 5 years. Respondents who did so were sent questionnaires to screen for further eligibility. Patients who fulfilled the criteria were instantly directed to the online questionnaires, after providing informed consent for study participation.

### 2.4 Measures

#### 2.4.1 Demographic variables and cancer characteristics

Socio‐demographic features included age, gender, educational level and marital status. Medical characteristics concerned time since cancer diagnosis, cancer type (e.g., breast, skin), currently receiving treatment (yes/no) and type of cancer treatment (e.g., surgery, chemotherapy). Furthermore, information about history of depression and previous psychological help was collected. All variables were obtained with single self‐report questions.

#### 2.4.2 Need for care

The primary outcome was need for psychological care, and was measured with a single question: “Would you like to receive psychological help?” Answering options were “yes” and “no.”

#### 2.4.3 Depressive symptoms

Depressive symptoms were measured using the PHQ‐9: a widely used and reliable self‐report questionnaire with good psychometric properties in cancer populations (e.g., Hinz et al.^28^). The PHQ‐9 includes nine items reflecting DSM‐5 symptoms for Major Depressive Disorder.^21^ Patients were asked how often they were bothered by these symptoms in the past 2 weeks, ranging from 0 (*not at all*) to 3 (*nearly every day*). We used three operationalizations of depressive symptoms based on the PHQ‐9 scores.

*Severity of depressive symptoms*. Severity of depressive symptoms was operationalized by summing all nine items of the PHQ‐9 to a total depression score ranging from 10 to 27. Cronbach's alpha was 0.60.

*DSM‐5 diagnosis of depression*. The diagnostic algorithm of the PHQ‐9, based on the DSM‐5, was used to categorize patients as “non‐depressed” or “depressed.”^21^ Patients were categorized as “depressed” when they scored two or higher on five or more PHQ‐9 items (one or higher on suicidal ideation) and at least one of these items was sad mood or loss of interest.

*Affective symptoms*. The ratio of affective symptoms compared to the total depression score represented the type of depressive symptoms that patients report. The allocation of items to the affective domain was done with a principal component analysis with Varimax rotation. Four items formed one (affective) factor: depressed mood, loss of interest, feeling worthless and suicidal ideation, with a Cronbach's alpha of 0.72.

#### 2.4.4 Illness perceptions

We used items of the validated Brief Illness Perception Questionnaire—Dutch Language Version^29^ to assess four core perceptions of depressive symptoms: consequences, duration, personal control and treatment control. In the introduction, specific symptoms that patients endorsed in the PHQ‐9 were summarized and patients were asked to keep these problems in mind when answering the items: how much do these problems affect your life? (consequences); how long do you think these problems will continue? (duration); how much control do you feel you have over these problems? (personal control); how much do you think treatment can help with these problems? (treatment control). The items could be answered with an eleven‐point Likert scale, with higher scores indicating more endorsement of the perception. To measure the fifth core perception “identity,” we used one item which stated that the problems the patient indicated before (i.e., the specific depressive symptoms reported on the PHQ‐9) might point to a depression, and consequently asked patients to what extent they thought they had experienced depressive symptoms in the past weeks. Answering categories ranged from 0 (*not at all*) to 3 (*to a severe extent*).

### 2.5 Statistical analysis

Statistical analyses were performed in SPSS (version 26). Descriptive statistics were presented as means and standard deviations or counts and percentages. There were no missing data. Assumptions were checked using scatterplots and quantile‐quantile plots. The first three research questions were answered with correlation analyses. Only illness perceptions that significantly correlated with the operationalization of depressive symptoms and need for care were included in the mediation model. We corrected for multiple testing by dividing the alpha level of 0.05 by the total number of variables.

Mediation analyses were conducted according to the Preacher and Hayes framework,^30^ using the PROCESS macro (version 3.4).^31^ One mediation model was tested for each operationalization of depression separately. Covariates were included if they were significantly correlated to both the operationalization of depressive symptoms and need for psychological care. We used bootstrapping to examine indirect effects of depressive symptoms on need for care through illness perceptions. Based on the bootstrap samples, unstandardized coefficients and their confidence intervals (CIs) were estimated. Specifically, the percentile bootstrapping method was used, with 5000 bootstrapped samples. Moreover, when heteroscedasticity was considered present, we used the PROCESS option to perform our analysis based on standard errors being robust against heteroscedasticity. Estimates of direct effects, indirect effects, and (bootstrapped) CIs for these effects were provided by PROCESS. We corrected for testing multiple mediation models by dividing the alpha level of 0.05 by three, the number of mediation models.^34^ As a result, CIs of 98.33% (abbreviated to 98%) were used which were significant if they did not include zero.

## 3 RESULTS

### 3.1 Participants

We approached 2549 patients, of which 2228 patients were screened for eligibility (see Figure S1). Of 1759 patients who received a cancer diagnosis in the past 5 years, 268 (15.2%) patients showed moderate to severe levels of depressive symptoms (PHQ‐9≥10). Almost one fourth received psychological care at the time of inclusion and was thus excluded. Of the remaining 202 patients, 127 (62.9% of 202) gave informed consent and completed the questionnaire.

Table 1 shows patients' demographic and clinical characteristics. The average age was 61 years. Most patients were female, married and had medium level education. Breast cancer was the most prevalent cancer type, and two‐thirds of the patients had received surgery. One third was receiving active cancer treatment at the time of inclusion. Almost 30% of patients had a history of depression and almost half had received psychological care before. None of the demographic or cancer characteristics—for instance including history of depression, having previously received psychological care or current cancer treatment status—were significantly associated with both the indicators of depressive symptoms and need for care.

**TABLE 1:** Demographic and cancer characteristics (*N* = 127)

	*N* (%) or mean (SD)
Gender (female)	72 (56.7%)
Age (in years)	61 (12)
Education	
Low	31 (24.4%)
Middle	58 (45.7%)
High	37 (29.1%)
Unknown	1 (0.8%)
Employment	
Retired	46 (36.2%)
Paid job	30 (23.6%)
Inability to work	27 (21.3%)
Doing the household	15 (11.8%)
Other^a^	9 (7.0%)
Partner status (%)	
Married or registered partnership	82 (64.6%)
Single	19 (15.0%)
Living together	10 (7.9%)
Other^b^	16 (12.5%)
Cancer type (multiple cancer types possible)	
Breast cancer	31 (24.4%)
Skin cancer	25 (19.7%)
Male genital cancer	16 (12.6%)
Digestive system cancer	12 (9.4%)
Urinary tract cancer	10 (7.9%)
Other^c^	40 (31.5%)
Cancer treatment (multiple treatments possible)	
Surgery	82 (64.6%)
Radiotherapy	44 (34.6%)
Chemotherapy	46 (36.2%)
Hormonal therapy	31 (24.4%)
Immunotherapy	9 (7.1%)
Other	12 (8.4%)
Time since (last) diagnosis (in months)	23 (17)
Active cancer treatment^d^	
Yes	44 (34.6%)
No	61 (65.5%)
History of depression	
Yes	36 (28.3%)
No	91 (71.7%)
Previous psychological care	
Yes	65 (51.2%)
No	62 (48.8%)

^a^ Including searching paid work, receiving education, being incapacitated for work and doing voluntary work.

^b^ Including widow/widower, divorced and having a partner but not living together.

^c^ Including respiratory tract, female reproductive organs, hematology, endocrine, head/neck, central nervous system and sarcoma.

^d^ Included hormonal therapy, radiotherapy, immunotherapy or chemotherapy.

### 3.2 Descriptive results

Table 2 shows the means and standard deviations or counts and percentages of the three operationalizations of depressive symptoms, illness perceptions and need for psychological care. The mean depression score was 14 (SD = 4.2), with 50.4% of patients classified as depressed. On average, 36% of depressive symptoms was attributed to affective symptoms. Thirteen percent indicated a need for psychological care.

**TABLE 2:** Descriptive results and correlations among depressive symptoms, illness perceptions and need for care (*N* = 127)

	Mean (SD) or *N* (%)	1	2	3	4	5	6	7	8	9
1. Severity of depression	14.39 (4.22)	‐								
2. DSM diagnosis	64 (50.4%)	0.65*	‐							
3. Affective symptoms	0.36 (0.13)	0.29*	0.44*	‐						
4. Consequences	6.92 (1.74)	0.39*	0.31*	0.19	‐					
5. Duration	6.89 (2.22)	0.18	0.11	0.05	0.39*	‐				
6. Personal control	4.76 (2.32)	−0.18	0.00	0.01	−0.29*	−0.13	‐			
7. Treatment control	4.31 (2.57)	0.11	0.11	0.04	0.17	−0.07	0.06	‐		
8. Identity	0.95 (0.79)	0.35*	0.30*	0.39*	0.29*	0.18	−0.08	0.20	‐	
9. Need for care	17 (13.4%)	0.02	1.61^a^	0.08	0.07	0.03	0.00	0.40*	0.26*	‐

^a^ Chi‐square value. We performed a chi‐square test because both variables are dichotomous.

*p ≤ 0.0056 (corrected for multiple comparisons).

### 3.3 Bivariate associations

None of the operationalizations of depressive symptoms were significantly associated with need for care (see Table 2). Severity of depression and DSM‐5 diagnosis showed significant positive correlations with perceived consequences and identity. Patients with higher levels of depressive symptoms and patients who met the DSM‐5 criteria of depression, perceived more consequences and more often identified their symptoms as depressive symptoms. The ratio of affective symptoms only significantly related to the identity dimension: patients with relatively more affective symptoms more strongly identified their symptoms as depressive symptoms. Of all illness perceptions, only treatment control and identity showed a significant positive association with need for psychological care. Stronger belief in treatment efficacy and higher identification of symptoms as being depressive symptoms related to higher perceived need for psychological care.

### 3.4 Mediation analyses

Identity was the only included mediator, since this was the only variable significantly related to the operationalizations of depressive symptoms as well as need for care. We tested three mediational models, for each operationalization of depressive symptoms separately. The models did not include covariates since none of the participant characteristics variables (see Table 1) significantly related to both the operationalization of depressive symptoms and need for care.

**FIGURE 1:**
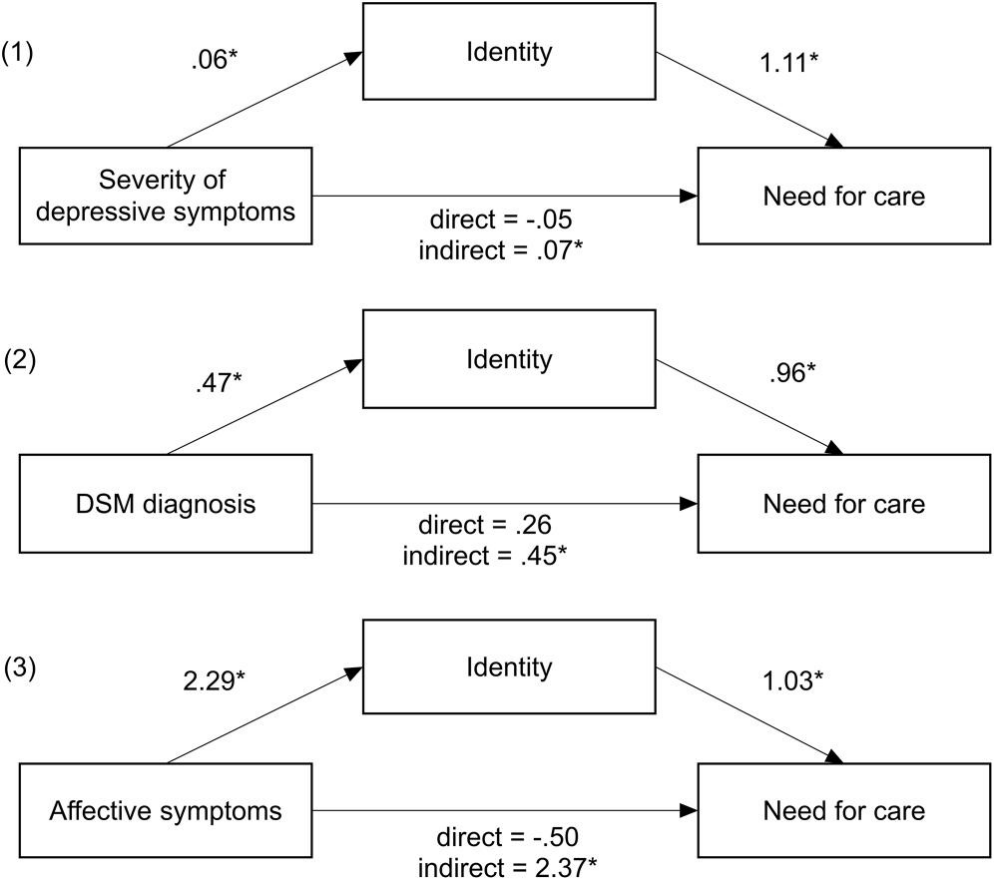
Simple mediation models for the three operationalizations of depression. The given numbers represent unstandardized coefficients. *p < 0.02

None of the three operationalizations of depressive symptoms significantly related to the need for psychological care (see Figure 1) (Severity: *B* = −0.05, SE = 0.07, 98% CI [−0.21, 0.10]; DSM‐5 diagnosis: *B* = 0.26, SE = 0.26, 98% CI [−1.09, 1.60]; Affective symptoms: *B* = −0.50, SE = 2.23, 98% CI [−5.68, 4.68]). However, all three operationalizations did significantly and positively relate to the need for psychological care via the identity dimension (Severity: *B* = 0.07, SE = 0.04, 98% CI [0.01, 0.18]; DSM‐5 diagnosis: *B* = 0.45, SE = 0.1.10, 98% CI [0.03, 1.23]; Affective symptoms: *B* = 2.37, SE = 1.10, 98% CI [0.36, 5.62]). Higher severity of depressive symptoms, being depressed according to the DSM‐5 criteria and experiencing a higher percentage of affective symptoms all led to a stronger identification of symptoms as being depressive symptoms. This in turn led to higher odds of perceiving a need for psychological care.

## 4 DISCUSSION

The current study was the first to examine depressive symptom perceptions and how these relate to need for psychological care in cancer patients with elevated levels of depressive symptoms who did not yet receive psychological care. Based on the Common Sense Model,^11^ we hypothesized a mediating role of patients' perceptions in the relationship between the three operationalizations of depressive symptoms and the need for psychological care. Results showed no direct associations between any of the operationalizations of depressive symptoms and need for psychological care. We did find a consistent significant and positive indirect effect of all operationalizations of depressive symptoms on the perceived need of care through the perception of identity.

First, depressive symptoms did not significantly relate to perceived need for care. Although current literature shows mixed results,^24^, ^32^ our findings add to the increasing body of literature showing that severity and diagnosis of depression are not predictive of perceived need for psychological care.^24^, ^25^, ^26^ Unexpectedly, experiencing relatively more affective symptoms also did not directly relate to need for care. Until now, only one study with diabetes patients found a positive significant association between the level of cognitive‐affective symptoms and perceived need for care, although this link was rather weak.^22^

Even though depressive symptoms and perceived need for psychological care did not relate directly, we did find a significant indirect relation via the identity perception. Patients with more severe symptoms of depression, who met the DSM‐5 criteria for depression, or who experienced relatively more affective symptoms more strongly related their symptoms to depression, and subsequently indicated a higher need for psychological care. This is in line with previous studies^33^, ^34^, ^35^ and implies that recognizing symptoms as depressive symptoms is important in help‐seeking. Improving the recognition of depressive symptoms might therefore be effective in increasing perceived need for psychological care.

Another relevant finding is that patients who perceived treatment as helpful, experienced a higher need for psychological care. This is in line with previous studies showing that positive attitudes towards psychological help predict intentions to seek psychological help.^15^, ^27^, ^36^ It might be beneficial to improve patients' negative perceptions about the efficacy of psychological treatment to increase psychological care needs. Future research could include broader measures of attitudes towards mental health problems and services to examine how these act as barriers in the uptake of psychological care.

Additionally, patients with more severe symptoms of depression or who met the DSM‐5 criteria perceived their symptoms as having more consequences on daily life, which is in accordance with previous research.^15^ Experiencing relatively more affective symptoms was not associated with perceiving more consequences during daily life, showing that perceived consequences might be mostly influenced by symptom severity, and not by symptom type, but this should be examined further in future research. Perceiving more consequences was not related to higher perceived need for care in our study. Previous research did find this relation in primary care patients,^27^ but our sample of cancer patients might have been more focused on their physical recovery^37^ than on possible beneficial effects of psychological treatment.

A strength of this study is that the hypothesized model was based on a strong theoretical rationale (i.e., the CSM framework). Our results partly support this framework by showing that depressive symptoms were indeed associated with illness perceptions (particularly perceived consequences and identity). Future research could examine why other illness perceptions such as personal control and the perceived duration of symptoms were not strongly related to depressive symptoms or need for care. A second strength is that by considering different operationalizations of depression, we took into account depressive symptom heterogeneity and the somatic overlap of depression and cancer. Lastly, we used a personalized approach to measure illness perceptions based on the symptoms patients had indicated before.

### 4.1 Study limitations

A limitation of this study is that the cross‐sectional design did not allow us to make claims about the directionality of relationships. Although we based our hypothesized models on the well‐established CSM framework,^11^ future longitudinal studies could study causal effects and examine how the course of depressive symptoms develops in the long run, hereby also including long‐term survivors of cancer. A second limitation is that whilst we intended to measure a perceived need for care, patients might have interpreted our question (“Would you like to receive psychological support for your complaints?”) as a preference to receive care or as a real treatment offer. Considering the burden of using an extensive questionnaire, such as the Perceived Need for Care Questionnaire,^38^ and the fact that a single question has been used before,^24^, ^26^, ^39^ we chose to use a single question to assess need for psychological care. However, future research could use qualitative methods and patient‐feedback to examine patients' understanding of a single question in measuring perceived need for care to create a validated and unified measurement. Lastly, the percentage of patients who perceived a need for psychological care was low (13%). Although this can be considered a finding of our study, this outcome did withhold us from testing complex models. Future research could focus on obtaining a larger sample to test the independent contribution of each illness perception dimension in the need for psychological care.

### 4.2 Clinical implications

Although preliminary, the results of the current study have several clinical implications. First, the lack of a direct relation between depressive symptoms and need for psychological care shows that screening for merely depressive symptoms might not be effective in selecting patients who perceive a need for psychological support. Rather, including patients' identity and treatment control perceptions in screening might be more effective in identifying those with a need for care. Moreover, informing cancer patients about depression and possible treatment options might improve their recognition of depressive symptoms and knowledge regarding the beneficial effects of psychological treatment. This can be done through, for example, information campaigns or psycho‐education given by health care professionals during follow‐up medical appointments.

## 5 CONCLUSIONS

In conclusion, our study showed that the identification of symptoms as depressive symptoms and the perceived effectiveness of psychological treatments play a role in reporting a need for psychological care. Identification of depressive symptoms becomes more pronounced when patients have more severe symptoms, meet DSM‐5 criteria for depression, and experience relatively more affective symptoms.

## CONFLICT OF INTEREST

The authors have no potential conflicts of interest to report.

## Supporting information

Figure S1

## Data Availability

The data that support the findings of this study are available from the corresponding author upon reasonable request.
