# Supplementary figures and images for: Understanding care needs of cancer patients with depressive symptoms: The importance of patients' recognition of depressive symptoms
Source: Psychooncology. 2021 Aug 10;31(1):62–9. doi: 10.1002/pon.5779 (PMC9292500; doi:10.1002/pon.5779)

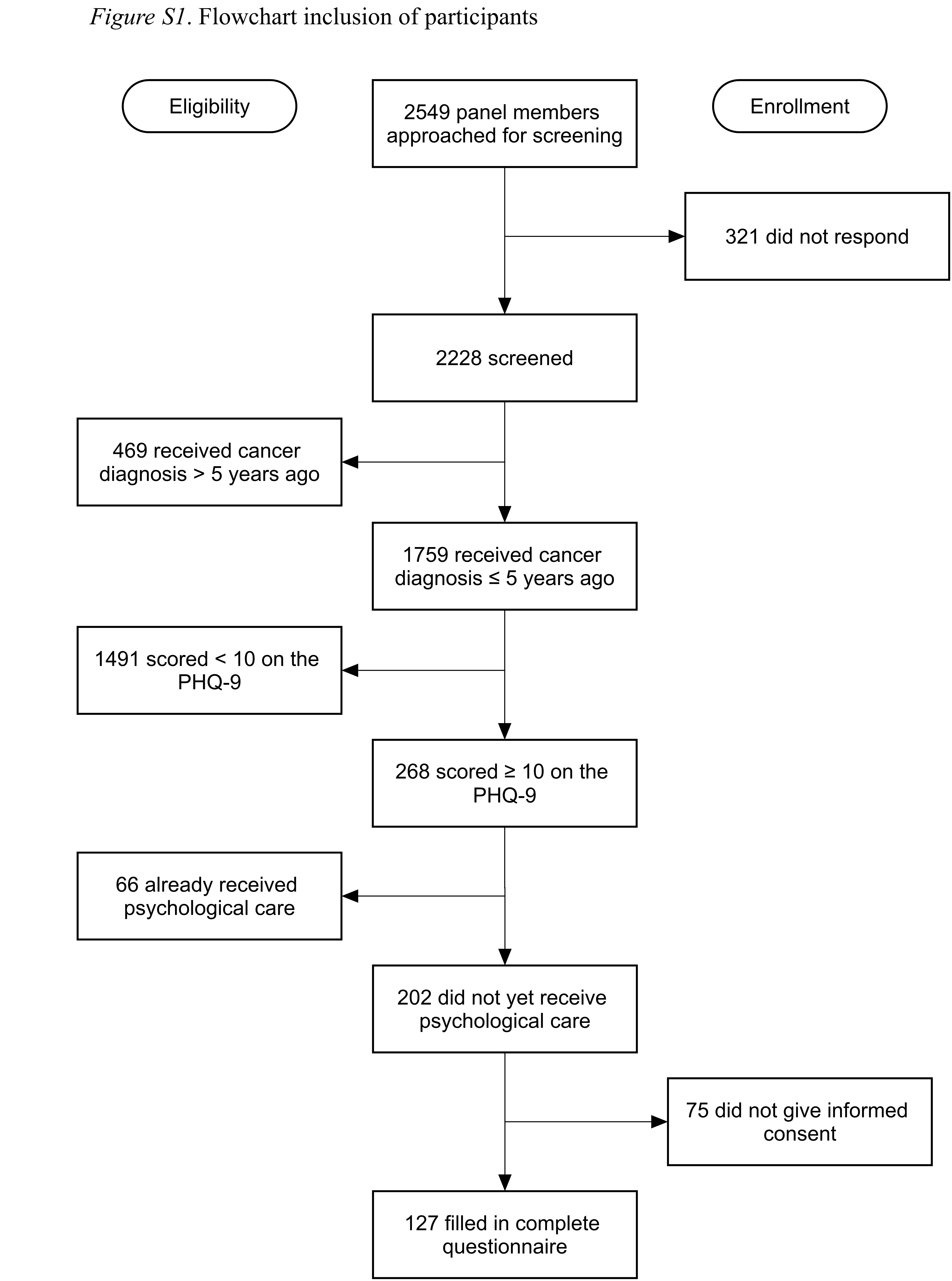

Supplement: Supplementary file 1 — Figure S1 [file PON-31-62-s001.png]
